# Fast Pyrolysis Bio-Oil-Based Epoxy as an Adhesive in Oriented Strand Board Production

**DOI:** 10.3390/polym14061244

**Published:** 2022-03-19

**Authors:** Osei Asibe Asafu-Adjaye, Jason Street, Archana Bansode, Maria L. Auad, Maria Soledad Peresin, Sushil Adhikari, Terry Liles, Brian K. Via

**Affiliations:** 1 School of Forestry and Wildlife Sciences, Auburn University, Auburn, AL 36849, USA; soledad.peresin@auburn.edu (M.S.P.); brianvia@auburn.edu (B.K.V.); 2 Department of Sustainable Bioproducts, Mississippi State University, Starkville, MS 39762, USA; jts118@msstate.edu; 3 Department of Chemical Engineering, Auburn University, Auburn, AL 36849, USA; asb0062@auburn.edu (A.B.); mla0001@auburn.edu (M.L.A.); 4 Department of Biosystem Engineering, Auburn University, Auburn, AL 36849, USA; sza0016@auburn.edu; 5 Huber Engineering Corporation, Commerce, GA 30530, USA; terry.liles@huber.com

**Keywords:** epoxy resin, fast pyrolysis, bio-oil, oriented strand board (OSB)

## Abstract

The objectives of this study were to utilize bio-oil-based epoxy resin in oriented strand board (OSB) production and investigate the effect of bio-oil substitution in epoxy resin as an adhesive for OSB production. Bio-oil was produced by the fast pyrolysis (FP) process using southern yellow pine (*Pinus* spp.). Bio-oil-based epoxy resin was synthesized by the modification of epoxy resin with FP bio-oil at various substitution levels. Acetone extraction using a Soxhlet process indicated a superior cured reaction of bio-oil and epoxy resin at 20% bio-oil substitution. FTIR spectra corroborated the Soxhlet extraction with the removal of the epoxide peak signature within the cross-linked polymer. Images from the scanning electron microscopy suggested bulk phase homogeneity. OSB panels were tested according to ASTM D1037-12. The modulus of rupture (MOR), modulus of elasticity (MOE), internal bond strength, and water resistance (thickness swell and water absorption) properties of the OSB panels were feasible at bio-oil substitution up to 30% in the epoxy resin system.

## 1. Introduction

Lignocellulosic biomass is known to be a suitable replacement for fossil-derived chemicals through thermochemical conversion processes. Biomass sourced chemicals are “green” and could reduce environmental pollution. One of the thermochemical processes which have received a myriad of attention is pyrolysis. Biomass pyrolysis to obtain a liquid fraction known as bio-oil occurs under anoxic conditions at high temperatures (~450–500 °C). When the residence time is at or less than 2 s, the process is called fast pyrolysis [1]. Detailed characterization of fast pyrolysis process and products is well-documented [2,3,4,5]. Reactive organic compounds are found in biomass fast pyrolysis (FP) bio-oil, including phenolic monomers and oligomers, furans, carboxylic acids, ketones, etc. [4,6]. In wood composite (e.g., OSB) manufacturing, wood residues from an off-cut could be processed and converted into fast pyrolysis bio-oil to provide energy for heating and chemicals for adhesive synthesis.

Oriented strand board (OSB) is engineered with multiple rectangular-like thin-cut wood strands (or flakes), coated with thermoset adhesives and are pressed at a high temperature and pressure. OSB is like plywood in construction in that the core layers are oriented perpendicular to the surface layers. Currently, polymeric diphenylmethane diisocyanate (pMDI) and phenol formaldehyde (PF) adhesives are the major adhesives used in OSB production. pMDI has been used as a core resin and PF as a surface resin in OSB production to accelerate curing, as pMDI has a relatively low curing temperature requirement when compared to PF in wood panels [7]. Interestingly, pMDI has become a standard adhesive in OSB manufacturing, supplanting phenol formaldehyde (PF). This is in part due to formaldehyde emission concerns from PF as a potential carcinogen. However, pMDI is expensive. Modification of pMDI with low-cost bio-based polymers remains a challenge, as it reacts readily within minutes of modification due to its high reactivity to other functional groups. Profuse bubbling has been reported within minutes when pyrolysis bio-oil was blended with pMDI for OSB production [8].

Epoxy (EP) resins are multipurpose thermosetting polymers used in construction, wood repairs, automobile parts, insulation materials, coatings, and paints owing to their high-strength properties, chemical resistance, good compatibility with other materials, good gap filling, and high thermal stability [9,10]. Nonetheless, EP resins are only used minimally as a wood adhesive in the wood composite industry. A major disadvantage of EP resin is cost and brittleness, requiring improvement [11,12]. Recent studies have focused on utilizing bio-oil to either physically blend with epoxy or synthesized epoxy resin. The results from these findings suggested that the hydroxyl groups found in bio-oil reacted with the epoxide groups to form cross-linked copolymer network structures [13,14,15,16]. Comparative lap-shear strength of bio-oil-based epoxy resins to commercial grade epoxy resin has also been reported using southern pine (loblolly) as a substrate [17].

The application of epoxy and epoxy modified with pyrolysis bio-oil in OSB production was the focus of this research. This work pioneers the use of epoxy and epoxy substituted with bio-oil in oriented strand board production. The study also characterized the hydroxyl groups (OH) of the bio-oil using quantitative ^31^PNMR, as the proportion of epoxy to OH groups is essential to epoxy cross-linking [18]. The chemical interaction of the modified epoxy-bio-oil was studied using FTIR. The thermal and chemical stability of the cured epoxy system was also analyzed. Thus, the objectives of this research were to utilize bio-oil-based epoxy resin in OSB production using southern yellow pine (a predominant species currently used in southern USA for OSB production) and investigate the mechanical and physical properties of OSB panels produced from the epoxy-bio-oil resin.

## 2. Materials and Methods

Norbord Inc. in Lanett, AL, USA, donated pre-screened pine wood flakes having an 8% moisture content. Epoxy resin (Epon 828) was purchased from Hexion Inc. (Columbus, OH, USA) Emulsified wax (Hexion Bord’N-Seal FMH-XD) and pMDI (MONDUR 541) were donated by J.M. Huber, Corporation (Commerce, GA, USA). Polypropylene glycol-based polyetheramine, a curing agent (JEFFAMINE T-430 ), was donated by the Huntsman Corporation, Woodlands, TX, USA. All chemicals were used as received and were of reagent grade.

### 2.1. Fast Pyrolysis (FP) Bio-Oil Production

Fast pyrolysis bio-oil from southern yellow pine wood was produced at Mississippi State University (Starkville, MS, USA) in a 7 kg h^−1^ auger-fed pyrolysis reactor [19]. The auger speed was 10 rpm at an applied pyrolysis temperature of 450 °C. The retention time was approximately 2 s. FP bio-oil was filtered to remove char particles using #1 Whatman paper. The main cross-linking reaction was hypothesized to occur between the hydroxyl (OH) moieties in the bio-oil and the epoxide in the epoxy resin. Therefore, ^31^P-NMR was used to quantitatively determine the categories of OH moieties in the FP bio-oil following the procedure [13] reported. Volumetric Karl Fischer titrator (Mettler Toledo, model V20, Columbus, OH, USA) with a hydranal-composite 5 solution (Sigma-Aldrich, St. Louis, MO, USA) was used for the bio-oil moisture content analysis.

### 2.2. Epoxy-Pyrolysis Bio-Oil Resin Formulation

The epoxy resin was heated to 65 °C on a water bath and the bio-oil was added to ensure uniform mixing for 30 min at 2000 rpm. The mixture was kept at 65 °C. Epoxy resins and their physical blends at different bio-oil substitution levels (Table 1) were hand-mixed with calculated amounts of Jeffamine T-403, *M_J_*, based on the epoxy equivalent weight (*EEW*), as in Equation (1):

(1) $$M_{J}=\frac{M_{E\;}\times AHEW}{\left(EEW\right)}\;$$

where *M_E_* is the mass of epoxy resin, and *AHEW* is the hardener amine hydrogen equivalent weight.

The viscosity of the adhesives was measured by a Fungilab rotary viscometer (Smart Series H, Model V210001, Hauppauge, NY, USA). The formulated adhesive was christened EP-bio-oil for the OSB production. Ten grams of epoxy resin and EP-bio-oil were respectively mixed with 10% acetone based on the epoxy solids for uniform mixing and were transferred into aluminum pans. The adhesives were cured in a heated conventional oven for resin characterization. The curing temperature was at 80 °C for 2 h, ramped to 120 °C for 1 h, and finally increased to 180 °C for an additional 1 h. The samples were kept in the oven until it reached room temperature.

### 2.3. FTIR Analysis

Fourier transform infrared (FTIR) spectra of FP bio-oil, neat epoxy resin, cured epoxy resin, and EP-bio-oil were obtained at a range of 4000 and 650 cm^−1^ with an FTIR spectrometer (Model Spectrum 400, Perkin Elmer Co., Waltham, MA, USA). The functional groups were determined at 64 scans with a 4.00 cm^−1^ resolution.

### 2.4. Soxhlet Extraction

The cured epoxy and epoxy-substituted bio-oil at different bio-oil inclusion rates were ground to a 40-mesh size. The Soxhlet extraction was achieved with a continuous 150 mL acetone reflux for 6 h as the unreacted bio-oil and epoxy dissolved in the acetone. Following the extraction, the remaining unextracted polymer was dried at 105 °C. The mass loss (%) for each adhesive formulation was estimated as a percentage by deducting the weight of unextracted polymer from the initial ground epoxy polymer.

### 2.5. Scanning Electron Microscopy (SEM) Analysis of Cured Epoxy and Epoxy-Bio-Oil

Cross-sectional surface morphology of the notched impact fractured cross-linked epoxy systems was observed using a Zeiss Merlin FE-SEM (Carl Zeiss Microscopy, Jena, Germany). The cross-linked epoxy systems were gold-coated prior to SEM analysis.

### 2.6. Fabrication of OSB

Oriented stand boards were manufactured following the method reported in [20] and the material parameters in Table 1. In a typical OSB batch fabrication, wax, followed by either pMDI or EP-bio-oil or epoxy, was sprayed onto the wood strands in a rotating blender equipped with a tumbler. The resin-coated strands were hand-oriented on a 430 × 430 mm forming box and then hot-pressed with a Wabash hydraulic press (model 50-24-2TM, Wabash, IN, USA) for 200 s (including closing and opening of the press) at constant temperature and pressure at 200 °C and 2 MPa, respectively. The desired board thickness of 11 mm was controlled with a distance bar placed at each side of the sandwiched platen. Panels made with pMDI adhesive only were used as the control. The anticipated density was 641 kg/m^3^. A total of 36 panels (6 panels each for each adhesive treatment) were produced. Half of each adhesive treatment panel was post-treated in an oven immediately after hot-pressing at 160 °C for 2 h following [21] for resins that may partially cure or may need a longer temperature exposure time. The moisture content for the wood strands was approximately 6 ± 1% prior to adhesive loading.

### 2.7. OSB Characterization

Test specimens were cut according to ASTM D1037-12 and conditioned at 20 ± 2 °C for seven days in a conditioning chamber at a relative humidity of 65%. The MOE, MOR, internal bond strength (IB), water absorption, and thickness swell (TS) of the test specimens were estimated following ASTM D1037-12 (ASTM International, 2012) recommendations.

### 2.8. Static Bending Test

The flexural properties (modulus of elasticity (MOE) and modulus of rupture (MOR)) of the OSB were determined using the three-point static bending test. Twelve specimens each of dimensions 305 × 76 mm were randomly selected from six panels each of the different adhesive formulations (six replicates were used in the dry test condition and the other half in the wet test condition). Samples designated wet were soaked in water for 24 h at 22 ± 1 °C. The bending test was carried on a universal testing machine (Zwick/Roell Z010). The load/deflection curve was obtained from tensile loading applied at a speed of 7.874 mm/min. TestXpert^®^ II software was used to compute the MOE and MOR in Equations (1) and (2), respectively, where *P_max_* is the maximum load (N), *b* is the width of the specimen (mm), *d* is the thickness of the specimen (mm), *L* is the length of the span (mm), Δ*P*/Δ*y* is the slope of the straight-line section of the load/deflection curve (N/mm), *E* is the modulus of elasticity (GPa), and *R_b_* is the modulus of rupture (MPa).

(2) $$E=\frac{L^{3}}{4bd^{3}}\;\frac{\Delta P}{\Delta y}$$

(3) $$R_{b}=\frac{3P_{max}L}{2bd^{2}}$$

### 2.9. Internal Bond Strength

Internal bond strength (IB), which measures the adhesive bond strength, was conducted in the dry condition according to the ASTM D1037-12 standard method. Ten replicates of dimensions 50 × 50 mm were used for each panel type. The IB samples were glued with hot-melt adhesive onto an aluminum alloy block and tested on a universal testing machine (Zwick/Roell, Z010, Ulm, Germany). The average tensile loading perpendicular to the glued specimen surface was calculated from the following equation:

(4) $$\mathrm{IB}=\frac{\mathrm{Maximum}\;\mathrm{load}\;\left(N\right)}{\mathrm{Length}\;\left(\mathrm{mm}\right)\;\times \;\mathrm{Width}\;\left(\mathrm{mm}\right)}$$

### 2.10. Thickness Swelling (TS) and Water Absorption (WA)

Specimens of dimensions 152 × 152 mm were prepared for the TS and WA tests. Five replicates each of the different adhesive formulation panels were tested. The weights of the samples were measured before and after soaking. Prior to soaking, the average dimensions of the samples were taken (from the four sides of the test specimens, 25 mm inward from the edge) with a vernier caliper. The samples were soaked in water at 23 ± 1 °C for 24 h. The samples were drained and wiped with a paper towel and the weights and dimensions were recorded as described above. The TS was evaluated as the percentage of the original thickness to the swelled thickness. WA was calculated as the percentage of the original weight to the swelled weight.

### 2.11. Data Analysis

Mean values of the MOE, MOR, IB, TS, and WA properties were analyzed by Minitab^®^ 19.1.1 (64-bit) software using a one-way analysis of variance (ANOVA). The different levels of FP bio-oil substitution and the effects of post-treatment were compared using Tukey’s honestly significant difference test (HSD) (*p* < 0.05).

## 3. Results

### 3.1. Hydroxyl (OH) Group Characterization of FP Bio-Oil: ^31^P-NMR Analysis

**Figure 1:**
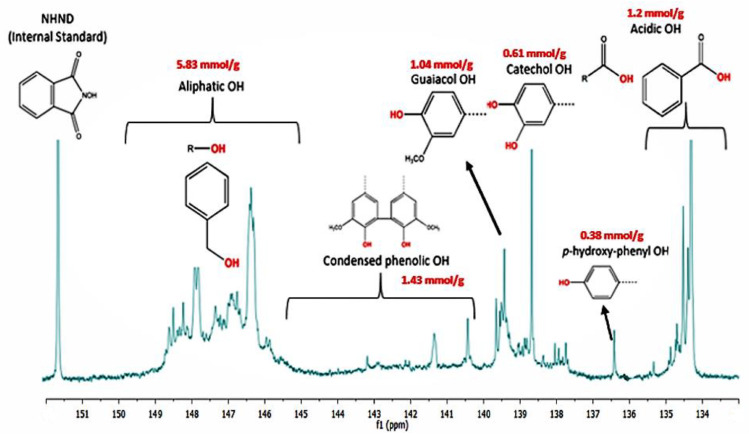
^31^PNMR analysis of fast pyrolysis bio-oil.

The total OH content of the FP bio-oil was 10.49 mmol/g. Detailed sources of the OH groups are shown in Figure 1. ^31^P-NMR of the FP bio-oil showed the presence of aliphatic and acidic OH groups which were generated as a result of holocellulose decomposition [22]. Phenolic OH such as C5-substituted condensed OH, p-hydroxy-phenyl OH catechol-type OH, and guaiacyl phenolic OH were also identified and were ascribed to depolymerization of lignin during pyrolysis [23].

### 3.2. FTIR Analysis

**Figure 2:**
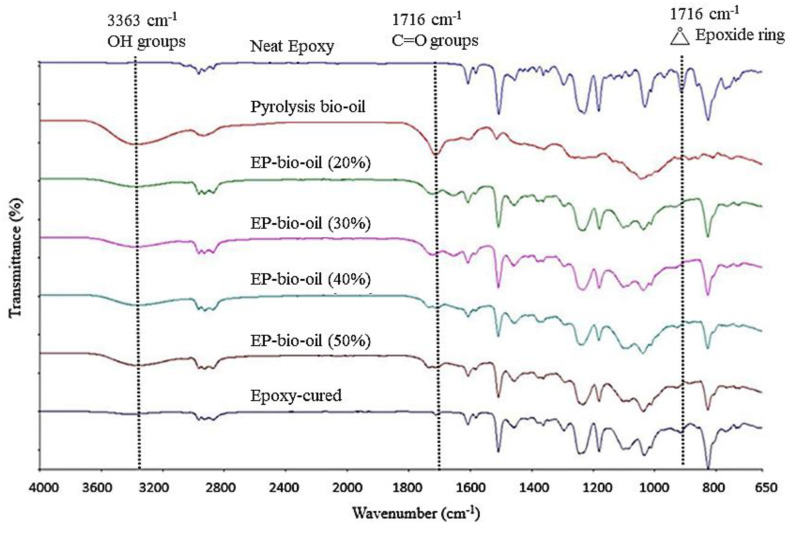
FTIR spectra of fast pyrolysis bio-oil, neat epoxy resin (Epon 828), cured epoxy, and epoxy-substituted bio-oil at different bio-oil contents (EP-bio-oil).

The IR spectra of FP bio-oil, epoxy resin (EPON 828), and epoxy-substituted pyrolysis bio-oil are illustrated in Figure 2. The broad peak at around 3363 cm^−1^ indicated the presence of OH groups in the bio-oil. ^31^P-NMR analysis corroborated the FTIR results that bio-oil contains substantial aromatic- and aliphatic-type OH groups. The band assignment at 1716 cm^−1^ was ascribed to carbonyl (C=O) groups in the bio-oil [24]. The presence of carbonyl moieties in bio-oil is associated with the decomposition of cellulose and hemicellulose of the pine biomass [3]. The band absorbances at 2968 and 1460 cm^−1^ were C–H stretch vibration and C–H bend vibration, respectively. Aromatic moieties in the bio-oil had characteristic peaks at 1019–1180, 1213–1297, 1608, and 1508 cm^−1^ [25]. A distinguishing characteristic peak for epoxy was assigned at 913 cm^−1^ for the epoxide ring. The individual IR spectrum of the EP-bio-oil revealed that the bio-oil formed a cross-linked network with the epoxy and the curing agent. For instance, the bio-oil showed a strong OH peak at 3363 cm^−1^, however the peaks were almost removed in the EP-bio-oil spectra, indicating that O–H groups participated in the epoxy resin curing.

On the other hand, the cured EP-bio-oil spectra showed a slight increase in the OH peak with high levels of bio-oil substitution. Interestingly, the epoxide group with the band assignment at 913 cm^−1^ was completely removed from the cured EP-bio-oil spectra. This confirmed that the bio-oil cross-linked with the epoxy and the curing agent. The IR spectra also revealed that the carbonyl groups (1716 cm^−1^) of the bio-oil also participated in the curing of epoxy resin, and hence a reduction in the carbonyl peak from the EP-bio-oil spectra. It could be inferred from the FTIR spectra that the hydroxyl and carbonyl groups within the pyrolysis bio-oil took part in the curing of the epoxy-bio-oil resin system. A possible epoxy reaction with bio-oil and fragment proposed in [17] in our lab is also proposed in this study.

### 3.3. Soxhlet Extraction

**Figure 3:**
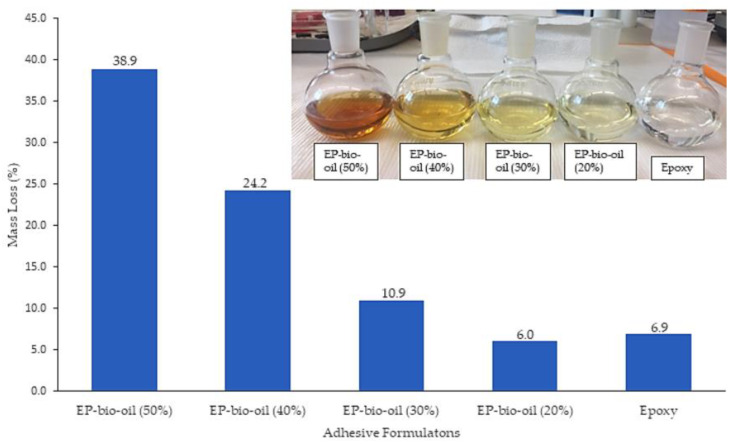
Mass loss (wt.%) of cured epoxy and epoxy-substituted bio-oil resin system under acetone extraction for 6 h.

Mass loss by acetone extraction of the cured polymer is shown Figure 3. Higher mass loss was associated with high levels of bio-oil substitution in the polymer matrix, an observation also seen in epoxy resin systems cross-linked with bio-oil using triphenylphosphine as a catalyst [13]. The less soluble product at 20% bio-oil substitution suggests a near stoichiometric ratio with a high cross-linked network polymer.

### 3.4. SEM of Cured Epoxy and Epoxy-Substituted Bio-Oil

**Figure 4:**
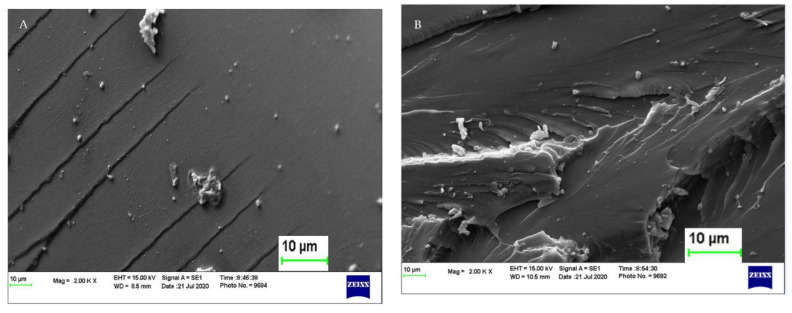
Morphology of cured epoxy samples by scanning electron microscopy. (**A**) Neat epoxy (left) and (**B**) epoxy-substituted bio-oil (right).

SEM analysis of the cured polymer is shown in Figure 4. Morphology of the cured epoxy resins exhibited comparable homogeneity. The fractured surface of the epoxy was smooth, indicating brittleness, whereas EP-bio-oil revealed an irregular fracture, suggesting an improved brittleness which may have resulted from the different chemical functionalities within the bio-oil matrix.

### 3.5. OSB Panel Characterization

The resin composition and viscosities of the different adhesive formulations are shown in Table 2. There was no significant difference between the panel densities (*p* < 0.05). The panel mat moisture content prior to hot-pressing increased with the bio-oil substitution. The one-way ANOVA indicated a significant difference (*p* < 0.05) among the OSB panels bonded with pMDI and the epoxy-bio-oil adhesive. The Tukey HSD test on the post-treated and untreated OSB panels for the bending properties, IB, TS, and WA, within each adhesive formulation was not significant (*p* < 0.05).

### 3.6. Bending Properties

**Figure 5:**
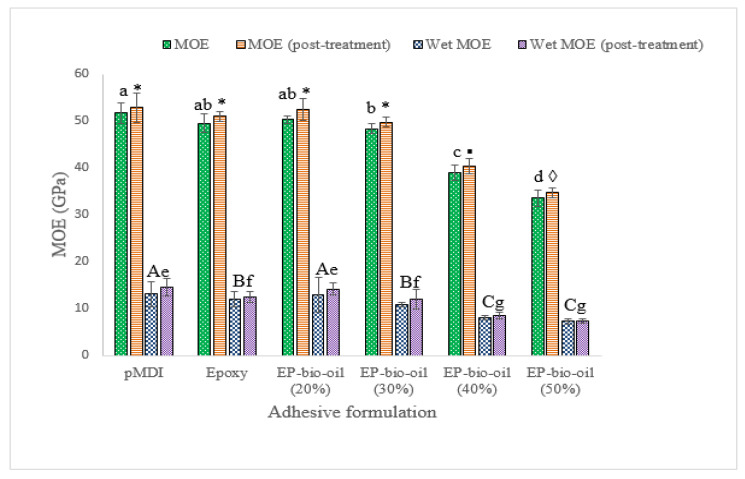
Modulus of elasticity (MOE) of OSB bonded with epoxy resin and epoxy-substituted bio-oil at 20%, 30%, 40%, and 50% bio-oil contents. Wet MOE was analyzed separately. EP-bio-oil (%): epoxy resin substituted with bio-oil. Different letters and symbols on the bar represent significant MOE (*p* < 0.05). Post-treated means were analyzed separately. Error bars represent standard deviation.

**Figure 6:**
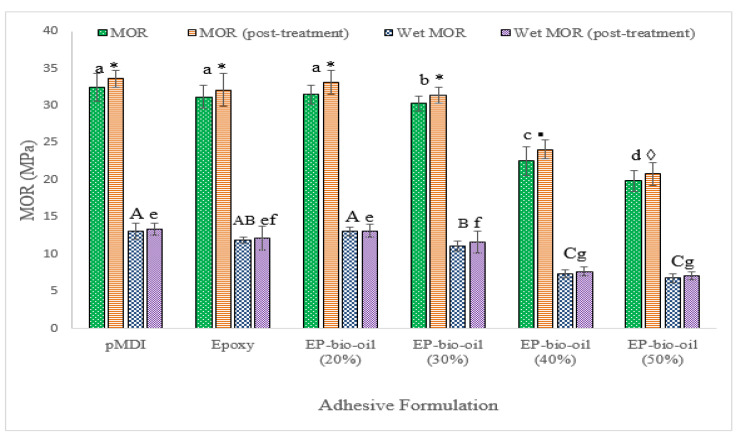
Modulus of rupture (MOR) of OSB bonded with epoxy resin and epoxy-substituted bio-oil at 20%, 30%, 40%, and 50% bio-oil contents. Wet MOR was analyzed separately. EP-bio-oil (%): epoxy resin substituted with bio-oil. Different letters and symbols on the bar represent significant MOR (*p* < 0.05). Post-treated means were analyzed separately. Error bars represent standard deviation.

The mean MOE and MOR values of the OSB panels are illustrated in Figure 5 and Figure 6. The MOE of panels made from 100% epoxy resin showed mean values of 49.6 ± 1.6 and 51.0 ± 1 GPa (post-treated). Substituting epoxy resin with FP bio-oil at 20% did not affect the MOE but compared favorably with pMDI (*p* < 0.05). A similar trend was observed in the MOR mean values regardless of the panel treatment. This may be ascribed to the optimal stoichiometric ratio of epoxy to bio-oil blend. However, a significant reduction in MOE and MOR at higher bio-oil substitution levels (>30%) was noted. The bending strength reduction at high bio-oil substitution is in part due to the high mat MC interfering with the epoxy-bio-oil curing and requiring a longer hot-pressing time to evaporate the moisture. For optimum cross-linking density to occur, the precise molar ratio of epoxy to hydroxyl groups’ blend is critical before curing [26]. It is probable that the higher amount of bio-oil replacement (>30%) may have deviated from the optimal stoichiometric ratio, leading to a reduction in cross-linking density and hence, a lower stiffness and lower MOR. A possible elucidation is that the FP bio-oil phenols and phenolic derivatives [27] could require a longer curing time in situ the wood panel matrix during hot-pressing.

Post-treatment improved the bending properties of the OSB panels within each adhesive formulation, although it was not statistically significant (*p* < 0.05). For example, the MOR of the untreated 20% bio-oil-substituted epoxy resin increased from 33.98 ± 2 to 35.58 ± 1 MPa when post-treated. It is worth mentioning that the MOE and MOR of the OSB panels bonded with neat epoxy resin were not significantly different from the epoxy substituted with 30% bio-oil.

### 3.7. Internal Bond Strength (IB)

**Figure 7:**
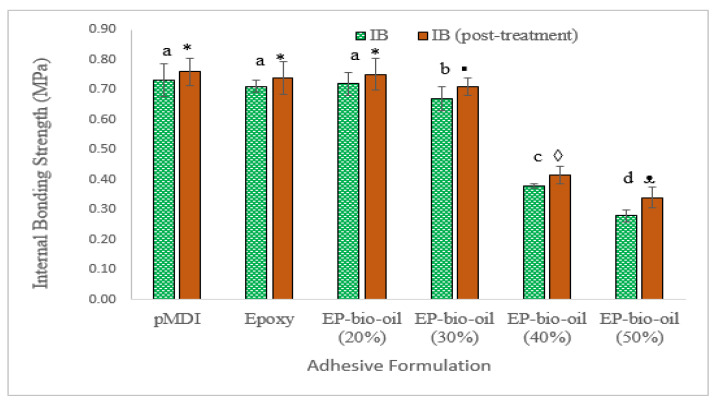
Internal bond strength of OSB bonded with the epoxy and epoxy-substituted bio-oil at 20%, 30%, 40%, and 50% bio-oil contents. EP-bio-oil (%): epoxy resin substituted with bio-oil. Different letters and symbols on the bar represent significant IB (*p* < 0.05). Post-treated means were analyzed separately. Error bars represent standard deviation.

The IB test results are shown in Figure 7. The IB strength of OSB samples showed a similar trend as seen in the bending properties. The bio-oil in this study had a substantial amount of water (~20%), which may have interfered with the adhesive bonding at high bio-oil substitution levels, and hence the low IB at high bio-oil substitution levels. Chan et al. observed a similar reduction in IB strength at high levels of pyrolysis bio-oil content with phenol formaldehyde resin [21]. As the bio-oil content increased in the epoxy resin system, the post-treatment effect on the IB also increased. For instance, the percentage increases in IB strength were ~10% and ~21% for bio-oil content of 40% and 50%, respectively, in the epoxy resin. It is likely that the improvement in IB strength of the panels after prolonged heating may have aided the reaction of residual epoxy resin with the bio-oil and the bulk wood panel. A longer hot-pressing time may be required to spread large bio-oil molecules on wood strand surfaces [8].

### 3.8. Thickness Swell (TS) and Water Absorption (WA)

**Figure 8:**
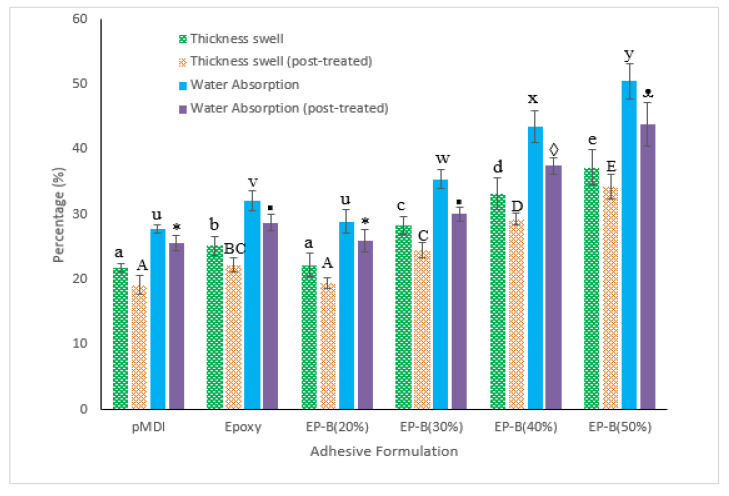
Thickness swell (TS) and water absorption (WA) bonded with the epoxy and epoxy-substituted bio-oil at 20%, 30%, 40%, and 50% bio-oil contents. EP-bio-oil (%): epoxy resin substituted with bio-oil. Different letters and symbols on the bar represent significant TS and WA (*p* < 0.05). Post-treated means were analyzed separately. Error bars represent standard deviation.

Figure 8 shows the 24 h water soak test. High levels of bio-oil (≥30%) in the epoxy resin system increased the TS and WA properties of the OSB panels. TS and WA increased by 68% and 75%, respectively, when bio-oil substitution in epoxy increased from 20% to 50%. It should be noted that lower values of TS and WA are preferred. Polar functional groups can form hydrogen bonding with the OH groups in wood [28]. At high bio-oil substitution levels, the unreacted bio-oil polar moieties, such as hydroxyls (OH) and carboxyls (COOH), may have formed hydrogen bonding with the OH of the wood strand’s surface. Nevertheless, water exposure readily disrupted such H bonds, thus lowering the OSB panel water resistance properties at high bio-oil content. The post-treated OSB specimens were relatively hydrophobic compared to the untreated samples due to the heat treatment, which could reduce water uptake and swelling of wood cell walls [29,30]. The prolonged heating by the post-treatment of samples may have removed free water resulting from the bio-oil, and to some extent, bound water from the wood cell walls. This may have improved the TS and WA in the post-treated samples.

## 4. Conclusions

Epoxy resin and epoxy substituted with bio-oil at various contents were successfully incorporated into the wood strands, and OSB boards were fabricated. The OH and C=O moieties in the bio-oil participated in the cross-linked network of the bio-oil-based epoxy resin system. The reduced mass loss by acetone extraction at 20% bio-oil substitution in the epoxy resin suggested a superior cross-linked polymer network. The mechanical and water resistance properties of the OSB from the epoxy resin systems revealed that a lower dosage of bio-oil up to a 30% substitution is feasible. A 20% bio-oil substitution was shown to improve the wet properties of the OSB panels and was comparable to pMDI. This study showed that the consumption of petrochemicals in OSB production can be reduced by utilizing pyrolysis bio-oil-amended epoxy resin.

## Figures and Tables

**Table 1:** OSB manufacturing parameters.

Item	Description
Adhesive loading	4% (oven dry wood basis)
Adhesive type used	Epoxy substituted with pyrolysis bio-oil; pMDI as control
Wax	1% (on oven dry wood basis)
Press temperature	210 °C
Post-treatment	160 °C for 2 h
Panel dimension	430 × 430 × 11 mm
Target density	641 kg/m³
Press time	200 s (closing and opening of press inclusive)
Epoxy/bio-oil substitution	80/20; 70/30; 60/40; 50/50

**Table 2:** Adhesive formulations with associated viscosities, panel density, and mat moisture content (MC %).

Resin Code	Epoxy (wt.%)	Bio-Oil (wt.%)	Viscosity (mPa s)	Panel Density (Kg/m^3^)	Mat MC (%)
PMDI	0	0	282	648 ± 2	8.4 ± 0.6
Epoxy (Epon 828)	100	0	472	647 ± 3	8.6 ± 0.8
EP-bio-oil (20%)	80	20	457	648 ± 3	9.2 ± 0.9
EP-bio-oil (30%)	70	30	443	647 ± 2	9.8 ± 0.7
EP-bio-oil (40%)	60	40	431	649 ± 4	10.6 ± 0.8
EP-bio-oil (50%)	50	50	416	646 ± 2	11.4 ± 0.5

## Data Availability

The data presented in this study are available upon request from the corresponding author.
